# Effects of light and darkness on pH regulation in three coral species exposed to seawater acidification

**DOI:** 10.1038/s41598-018-38168-0

**Published:** 2019-02-18

**Authors:** A. A. Venn, E. Tambutté, N. Caminiti-Segonds, N. Techer, D. Allemand, S. Tambutté

**Affiliations:** 1 Marine Biology Department, Centre Scientifique de Monaco, 8 Quai Antoine 1er, 98000 Monaco, Monaco; 2 Laboratoire Européen Associe 647 « BIOSENSIB », Centre Scientifique de Monaco- Centre National de la Recherche Scientifique, 8 Quai Antoine 1er, 98000 Monaco, Monaco

## Abstract

The resilience of corals to ocean acidification has been proposed to rely on regulation of extracellular calcifying medium pH (pH_ECM_), but few studies have compared the capacity of coral species to control this parameter at elevated pCO_2_. Furthermore, exposure to light and darkness influences both pH regulation and calcification in corals, but little is known about its effect under conditions of seawater acidification. Here we investigated the effect of acidification in light and darkness on pH_ECM_, calcifying cell intracellular pH (pH_I_), calcification, photosynthesis and respiration in three coral species: *Stylophora pistillata*, *Pocillopora damicornis* and *Acropora hyacinthus*. We show that *S. pistillata* was able to maintain pH_ECM_ under acidification in light and darkness, but pH_ECM_ decreased in *P. damicornis* and *A. hyacinthus* to a much greater extent in darkness than in the light. Acidification depressed calcifying cell pH_I_ in all three species, but we identified an unexpected positive effect of light on pH_I_. Calcification rate and pH_ECM_ decreased together under acidification, but there are inconsistencies in their relationship indicating that other physiological parameters are likely to shape how coral calcification responds to acidification. Overall our study reveals interspecies differences in coral regulation of pH_ECM_ and pH_I_ when exposed to acidification, influenced by exposure to light and darkness.

## Introduction

The dissolution of anthropogenic carbon dioxide in the world’s oceans is simultaneously reducing seawater pH and the concentration of carbonate ions, a process commonly termed “ocean acidification”. As ocean acidification intensifies in future decades and beyond, changes in ocean carbonate chemistry are predicted to have negative impacts for many marine organisms. Biomineralizing marine organisms, such as reef-building corals, have been highlighted as particularly vulnerable to ocean acidification, due to the deleterious effects of ocean acidification on both rates of calcification and skeleton formation^1,2^. Understanding this vulnerability and any potential resilience to future ocean acidification hinges on an improved mechanistic vision of the physiological processes driving calcification^3^.

In reef corals, the aragonite skeleton forms in the extracellular calcifying medium (ECM) (=subcalicoblastic medium), which is spatially separated from the external seawater environment by the overlying calicoblastic (calcifying) epithelium and other overlying tissue layers. Current knowledge of ECM chemistry is limited, but several studies have shown that the pH of the calcifying medium (pH_ECM_) is elevated with respect to the surrounding seawater. These include studies that have made direct measurements of pH_ECM_ by confocal microscopy using pH sensitive dyes^4–6^, studies that have inserted microelectrodes into the coral tissues^7–9^ and analysis of boron isotopes in the aragonite skeleton itself^10,11^. Because elevations in pH increase the relative concentration of CO_3_^2−^ in the total dissolved inorganic carbon (DIC) pool and thus the saturation state of aragonite (Ω) of the ECM, regulation of pH_ECM_ is considered to be one of the key steps in the biological control of biomineralization: setting up an elevated saturation state of aragonite in order to promote the calcification reaction^12^.

The importance of pH_ECM_ regulation in calcification physiology has led to intense research interest into how this parameter responds to ocean acidification. Studies have shown that pH_ECM_ declines in response to changes in seawater pH, potentially lowering Ω of the ECM which could be unfavourable to the maintenance of calcification rates^5,8^. However, research in the laboratory, field-based mesocosms and at natural CO_2_-vent sites have demonstrated that several coral species are capable of maintaining elevated pH_ECM_ despite severe decreases in external seawater pH^5,13,14^. Sometimes referred to as “pH upregulation” this trait has been suggested to be a possible physiological mechanism of resistance against ocean acidification^5,15^. However, there are surprisingly very few comparative studies that investigate the relative ability of different coral species to regulate pH_ECM_ under seawater acidification treatments. Previous comparisons of the capacity of corals to regulate pH_ECM_ against seawater acidification have mainly been conducted by compiling the results of different studies, which used different methodologies (e.g. boron isotopes, confocal microscopy)^15,16^. Few studies have directly compared the capacity of different coral species to regulate pH_ECM_ in a single controlled investigation apart from a recent study on *Pocillopora damicornis* and *Acropora youngeii*^17^.

Exposure to light or darkness is likely to be an important environmental factor in shaping the response of pH_ECM_ regulation to ocean acidification, as it is already known to affect coral calcification rates. Many corals are symbiotic with photosynthetic dinoflagellates (family *Symbiodiniaceae*^18^) and it is known that light can enhance calcification rates with respect to dark values (Light-Enhanced Calcification or LEC)^19,20^. More recently is has been shown that exposure to light can also influence the susceptibility of coral calcification rates to acidification^21,22^. Other work in the last decade has shown how light drives pH changes in coral endoderm intracellular pH (pH_I_) and how light-induced elevations in pH_I_ mitigate endoderm cell acidosis at elevated seawater pCO_2_^23–25^. However, there is currently little information on the effect of light and darkness on pH_ECM_ and intracellular pH (pH_I_) of the calicoblastic cells, particularly under conditions of acidification.

Here, we addressed these knowledge gaps in the current literature with a study that investigated how exposure to light and darkness affects pH_ECM_ and pH_I_ regulation in different species of symbiotic coral under seawater acidification. We selected three species of coral that would potentially display differential responses to acidification. Two of these species, *Stylophora pistillata* (which is widely considered as resistant to ocean acidification^26^) and *Pocillopora damicormis* (for which responses to pCO_2_ are variable), belong to the scleractinian “robust” clade^27^. The third species, *Acropora hyacinthus*, is a member of a genus that is generally considered as vulnerable to ocean acidification and is a member of the “complex” clade^28–30^. The investigation was conducted under controlled laboratory conditions, allowing us to carry out *in-vivo* confocal microscopy to analyse pH regulation in both calcifying cells and the extracellular calcifying medium under different levels of acidification in darkness and at one irradiance level. To perform confocal analysis measurements were conducted on coral microcolonies grown on glass coverslips. In parallel experiments, we also investigated the effect of seawater acidification on calcification, photosynthesis and respiration rates in the three species using microcolonies suspended from monofilament threads.

## Materials and Methods

### Experimental set-up

Colonies of *Stylophora pistillata*, *Pocillopora damicornis* and *Acropora hyacinthus* were grown in the long-term coral culture facilities at the Centre Scientifique de Monaco where aquaria were supplied with flowing seawater from the Mediterranean sea (exchange rate 2% h^−1^), at a salinity of 38, under an irradiance of 175 µmol photons m^−2^ s^−1^ of photosynthetically active radiation (PAR) (400–700 nm) on a 12 h: 12 h light: dark cycle. Samples were prepared from mother colonies as nubbins suspended on monofilament threads (for measurements of calcification, photosynthesis and respiration) or microcolonies grown laterally on glass coverslips (for *in vivo* confocal microscopy of pH_ECM_ and pH_I_ as described previously^4^). Temperature was monitored continuously by temperature sensors (Ponsel, France) and a monitoring system (Enoleo, Monaco) and maintained at 25 ± 0.3 °C (mean ± SD). Corals were fed daily with frozen rotifers and twice weekly with live *Artemia salina* nauplii.

For exposure to seawater acidification, colonies were transferred from the long-term culture facilities described above to eight seawater acidification aquaria supplied with Mediterranean seawater (exchange rate 60% per hour), at the same salinity, temperature and under the same irradiance conditions described above for culture facilities. This seawater acidification setup has been in continuous operation for several years and has been described in previous publications^2,31^. Each pH treatment was represented by two aquaria and coral colonies were randomly distributed as evenly as possible between them. Exposure to seawater acidification was conducted for a one-week duration. That is to say that each coral used in the investigation was transferred from long-term culture tanks to a tank in the seawater acidification set up for 1 week, before being removed and analysed for the appropriate physiological parameter. Because the number of replicates meant that all corals could not be analysed with in a single week, one week exposures in treatment tanks were repeated in time for 6 months to gather all the necessary data.

### Control and maintenance of carbonate chemistry of the seawater pH treatments

In 6 of the 8 aquaria, carbonate chemistry was manipulated by bubbling with CO_2_ to reduce pH aquaria to the target values of pH_T_ 7.2, 7.4 and 7.8 (Table 1). The two other aquaria were bubbled with CO_2_- free air (Tanks 1 and 2, pH 8.1, Table 1). Submersible pumps (EHEIM 3000, pump power 1800 l/h) ensured high water circulation in each aquarium. Aquaria were rigorously cleaned every week to prevent the growth of epiphytic algae and fouling communities or the accumulation of detritus.

**Table 1:** Carbonate chemistry parameters in the four experimental pH treatments.

Treatment	Tank	TA (µmol/kg-SW)	pH_T_	TC (µmol/kg-SW)	pCO_2_ (µatm)	HCO_3_^−^ (µmol/kg-SW)	CO_3_^2−^ (µmol/kg-SW)	Ω*ar*
pH 8.1	1	2438.57	8.03	2105.15	415.91	1856.63	236.93	3.68
		± 9.81	± 0.01	± 2.94	± 8.99	± 1.62	± 4.81	± 0.07
pH 8.1	2	2433.70	8.03	2100.58	414.72	1852.48	236.55	3.67
		± 8.45	± 0.01	± 1.59	± 9.44	± 2.90	± 4.75	± 0.07
pH 7.8	3	2438.43	7.83	2221.28	725.37	2037.47	163.60	2.54
		± 6.69	± 0.01	± 1.83	± 14.58	± 0.96	± 3.20	± 0.05
pH 7.8	4	2437.13	7.79	2242.44	816.67	2069.73	149.95	2.33
		± 6.93	± 0.01	± 3.39	± 11.51	± 1.37	± 2.35	± 0.04
pH 7.4	5	2458.32	7.45	2401.39	1923.08	2271.13	76.67	1.19
		± 5.72	± 0.02	± 1.09	± 80.61	± 2.05	± 3.21	± 0.05
pH 7.4	6	2459.39	7.42	2415.76	2104.62	2286.13	70.99	1.10
		± 5.67	± 0.01	± 1.42	± 54.32	± 0.99	± 1.94	± 0.03
pH 7.2	7	2462.29	7.25	2479.63	3172.89	2341.81	49.41	0.77
		± 6.83	± 0.01	± 2.72	± 77.57	± 3.49	± 1.39	± 0.02
pH 7.2	8	2463.76	7.22	2490.73	3378.78	2349.87	46.72	0.73
		± 5.76	± 0.01	± 2.20	± 71.13	± 3.05	± 1.13	± 0.02

(Means ± SD). TA = Total alkalinity. TC = total dissolved inorganic carbon. Parameters of carbonate seawater chemistry were calculated from measured total scale pH, TA, temperature (25 °C), and salinity 38.

pH electrodes (Ponsel-Mesure, France) calibrated to pH total scale and temperature sensors (Ponsel, France) were installed in the tanks and connected to a custom-made monitoring system (Enoleo, Monaco) which monitored pH and temperature continuously, and controlled CO_2_ bubbling rates and heating elements. pH measurements were also made using the indicator dye m-cresol purple (Acros 199250050) adapted from Dickson *et al*.^32^; the absorbance was measured using a spectrophotometer (UVmc^2^; Safas, Monaco). Measurements of total alkalinity (TA) were made according to protocols described in Dickson *et al*.^32^. TA was measured via titration with 0.03 N HCl containing 40 g NaCl l^−1^ using a Metrohm Titrando 888 Dosimat controlled by Tiamo software to perform automated titrations of 4-mL samples, and alkalinity was calculated using a regression routine based on Department of Energy guidelines^33^. For each sample run, certified seawater reference material supplied by the laboratory of A. G. Dickson (Scripps Institution of Oceanography, La Jolla, CA) was used to verify acid normality. Parameters of carbonate seawater chemistry were calculated from total scale pH, TA, temperature, and salinity using the free-access CO2SYS package^34^ using constants from Mehrbach *et al*.^35^ as refit by Dickson and Millero^36^. Spectrophotometric pH measurements and TA measurements were taken weekly, in addition to continuous monitoring by pH electrode during the 6-month period in which replicate week-long seawater acidification exposures were carried out. Mean values and standard errors of parameters of carbonate seawater chemistry in each treatment are given for the 6-month experimental period in Table 1.

### Confocal microscope measurements of extracellular calcifying medium pH and intracellular pH in calcifying cells

Measurements of pH of the extracellular calcifying medium (pH_ECM_) and intracellular pH (pH_I_) in calicoblastic cells in the light and dark were made on separate samples by inverted confocal microscopy (Leica SP5, Germany) and the ratiometric dye SNARF-1 (Invitrogen) according to methods we published previously^4,17^.

Samples grown laterally on glass coverslips were fitted in semi-closed perfusion chambers (PeCon, Germany) and mounted on the confocal microscope and supplied by perfusion with seawater drawn from the desired acidification treatment. A single irradiance level was provided at the same level as treatment aquaria (175 μmol photons m^−2^·s^−1^ PAR, which has also been used in previous investigations^2,37^), and temperature maintained at 25 °C. The chosen irradiance and temperature also corresponded to the light level provided during long-term culture of parent colonies and growth of the coral microcolonies and the acidification treatments.

A renewal rate of 50% per min of a 2.5-mL volume in the perfusion chamber ensured that seawater pH remained stable in both light and dark conditions^2^. The pH of the perfused seawater was checked by making confocal pH measurements in the seawater surrounding the corals to check that pH did not drift away from the target values used in treatments during the period of measurement (seawater pH values in the perfusion chamber are given in Supplementary 1). Measurement of oxygen in seawater in the perfusion chamber with a needle-type microsensor (PreSens, Germany) in light and darkness indicated oxygen levels also remained stable between values of 265–280 µmol l^−1^ under these conditions.

After being transferred from the treatment aquaria directly to the microscope, samples were first perfused with seawater from the desired experimental treatment for 20 min in either the light or dark. For pH_ECM_, samples were then perfused with seawater from the desired treatment containing 45 µM cell-impermeable SNARF-1 for a 5 min loading period, before making five measurements of pH_ECM_ during a 10 min time window in light or dark. pH measurements were also taken in the seawater surrounding the corals in the perfusion chamber to confirm seawater pH remained stable during confocal analysis.

For measurements of pH_I_ of calicoblastic cells, the procedure involved 10 min of dye loading by perfusion with seawater containing 10 mM cell-permeable SNARF-1 AM, followed by 10 min of seawater perfusion, during which pH_I_ measurements were taken to check pH_I_ was stable. Calibration of fluorescence of intracellular SNARF-1 AM and extracellular SNARF-1 (seawater and ECM) with pH was performed as described previously^4^ to the National Bureau of Standards (NBS) pH scale and total scale pH, respectively.

pH_ECM_ and calicoblastic cell pH_I_ measurements were carried out at 40X magnification by excitation at 543 nm at 30% laser intensity, and fluorescence captured at emission wavelengths of 585 ± 10 nm and 640 ± 10 nm. For each measurement, several optical sections were captured in a Z-stack without contamination by chlorophyll autofluorescence by the symbiotic algae in the overlying tissues^4,23^. pH_ECM_ was measured in light and dark conditions in 5 samples from each treatment. Calicoblastic cell pH_I_ was measured in 3 colonies from each treatment.

### Calcification

Calcification was measured using the alkalinity anomaly technique^38^ using microcolonies suspended from monofilament threads. After 7 d of incubation in each pH treatment, colonies were chosen randomly from the tanks and placed in separate 50-mL plexi-glass beakers containing filtered (0.2 µm) seawater from the respective treatment tanks. Incubations were performed in light or darkness for 1 h with the same conditions of temperature (25 °C) and light (175 µmol photons m^−2^ s^−1^) as in the treatment aquaria, after which 20 ml of seawater was removed for TA measurement (procedure described above). Alkalinity anomalies were calculated correcting for treatment-specific blank beakers and taking sample displacement volume into account. Calcification rates derived from alkalinity anomalies were normalized to surface area, total protein and skeletal mass (see below). Light and dark calcification rates were measured on three coral microcolonies per treatment in the case of *S. pistillata* and *P. damicornis*, and six microcolonies per treatment for *A. hyacinthus*.

### Photosynthesis and respiration rates and biomass parameters

Microcolonies were transferred directly from treatment aquaria in the light period to individual closed beakers. Each microcolony was suspended by its monofilament thread in the beaker and incubations were performed in the same conditions of temperature (25 °C) and seawater chemistry as in experimental tanks, but either under light conditions (175 μmol photons m^−2^ s^−1^) for photosynthesis, or dark conditions for respiration. Mixing was achieved with a magnetic stirrer and seawater pH and alkalinity were checked at the end of each experiment. An oxygen optode sensor system (oxy-4 mini, PreSens, Regensburg, Germany) was used to quantify oxygen flux. Data were recorded with OXY4v2_11FB software (PreSens). Before each measurement, the oxygen sensor was calibrated against air-saturated seawater (100% oxygen) and a saturated solution of sodium sulfite (zero oxygen). Rates of net photosynthesis and respiration were estimated by regressing oxygen data against time after an initial period of approximately five minutes in which rates were allowed to stabilize. Following analysis, samples were stored at −20 °C. Photosynthesis and respiration rates were derived taking sample displacement volume into account and normalized to skeletal mass. 5 coral microcolonies were measured per treatment.

### Analysis of protein, surface area and skeletal mass

Frozen samples were placed in 0.5 N NaOH and tissues removed with a jet of pressurized nitrogen. The tissue slurry was then incubated at 90 °C for 10 min. Protein content was then determined using the bicinchoninic acid assay kit (BC Protein Assay, Interchim). The standard curve was established with bovine serum albumin and the absorbance was measured with a microplate reader (EpochTM, Bioteck, US) at 562 nm.

Following removal of tissues, the skeleton was collected, rinsed first in tap water and then in distilled water, oven-dried for several days at 60 °C and then weighed to determine skeletal mass. These skeleton samples were then used for measuring surface area. Colony surface area was measured using one of the common methods currently used, the paraffin wax method^39^. Briefly, coral skeletons were coated in paraffin wax by dipping in Paraplast wax (Sigma, France) at 65 °C. Surface area of the specimens was obtained by referring the weight of the paraffin wax coated on the specimen to the standard curve of paraffin wax versus surface area. The standard curve was generated by regressing weight of the paraffin wax to known surface area density blocks.

### Statistical analysis

Data were analyzed using the programme SPSS v. 24 (IBM, France). Following Shapiro-Wilk’s tests and Levene’s tests to check the data adhered to the assumptions necessary for parametric analysis (normal distributions with homogenous variances), the parameters pH_ECM_, pH_I_ and calcification rate were analyzed by three-way ANOVA using species, seawater pH and light/darkness as independent variables. Photosynthesis and respiration rates were analysed by two-way ANOVA with seawater pH and species as independent variables. Posthoc analysis was carried out on significant main effects by Tukey tests. Significant interactions were analysed by simple effects analysis and pairwise comparisons with Bonferroni corrections. Results of ANOVAs are reported in Table 2. The results of simple effects analysis are reported in Table 2 and Supplementary 2.

**Table 2:** Results of three way ANOVAs for pH_ECM_, pH_i_ (Fig. 1) and calcification, and two way ANOVAs for photosynthesis and respiration (Fig. 2).

Variable	Effect	*df*	MS	*F*	*P*	Post hoc
pH_ECM_	pH_SW_	3.00	1.56	242.66	**<0.0001**	8.1 > 7.8, 7.4, 7.2; 7.8 > 7.4, 7.2; 7.4 > 7.2
	L/D	1.00	0.55	85.48	**<0.0001**	Light > Dark
	Sp	2.00	0.25	38.33	**<0.0001**	SP > PD, AH
	pH_SW_ * lightdark	3.00	0.02	2.70	0.05	
	pH_SW_ * species	6.00	0.02	2.48	**0.03**	See S 1
	lightdark * species	2.00	0.09	13.70	**<0.0001**	See S 1
	pH_SW_ * lightdark * species	6.00	0.02	3.34	**0.01**	See S 1
	Error	96.00	0.01			
pH_I_	pH_SW_	3	0.22	107.86	**<0.0001**	8.1 > 7.8, 7.4, 7.2; 7.8 > 7.4, 7.2; 7.4 > 7.2
	L/D	1	0.04	18.79	**<0.0001**	Light > Dark
	Sp	2	0.55	271.63	**<0.0001**	SP > PD; SP > AH; PD > AH
	pH_SW_ * lightdark	3	0.00	1.27	0.294	
	pH_SW_ * species	6	0.01	4.94	**<0.001**	See S 1
	lightdark * species	2	0.00	1.19	0.314	
	pH_SW_ * lightdark * species	6	0.00	0.25	0.955	
	Error	48	0.00			
Calcification rate	pH_SW_	3	8394.50	45.93	**<0.0001**	8.1 > 7.4, 7.2; 7.8 > 7.4, 7.2; 7.4 > 7.2; 8.1 = 7.8
	Light/Dark	1	57911.62	316.87	**<0.0001**	Light > Dark
	Species	2	16810.57	91.98	**<0.0001**	PD > SP; PD > AH; SP > AH
	pH_SW_ * lightdark	3	297.56	1.63	0.195	
	pH_SW_ * species	6	899.73	4.92	**<0.001**	See S 1
	lightdark * species	2	1803.86	9.87	**<0.0001**	See S 1
	pH_SW_ * lightdark * species	6	831.49	4.55	**<0.001**	See S 1
	Error	48	182.76			
Photosynthetic rate	pH_SW_	3	0.16	0.24	0.87	
	Species	2	7.49	11.22	**<0.0001**	AH > SP, PD
	pH_SW_ * Species	6	2.67	3.99	**<0.0001**	See results text
	Error	48	0.67			
	Total					
Respiration rate	pH_SW_	3	0.37016	0.649083	0.587392441	
	Species	2	6.42856	11.272649	**<0.0001**	SP > AH, PD
	pH_SW_ * Species	6	0.38002	0.6663842	0.677041204	
	Error	48	0.57028			

SP = *Stylophora pistillata*; PD = *Pocillopora damicornis*; AH = *Acropora hyacinthus*.

## Results

### Extracellular calcifying medium pH in light and darkness under acidification

**Figure 1:**
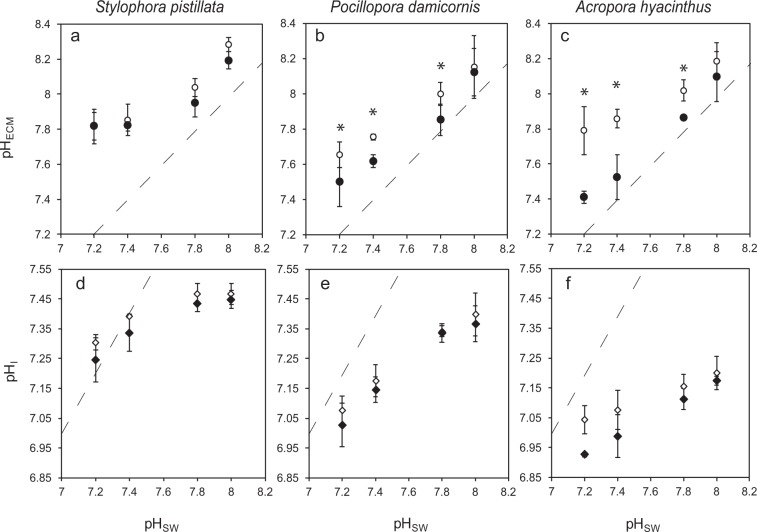
Effects of seawater acidification in light (open symbols) and darkness (closed symbols) on extracellular calcifying medium pH (pH_ECM_) (**a–c**) and calcifying cell pH (pH_I_) (**d–f**) in *Stylophora pistillata* (column **a–d**), *Pocillopora damicornis* (column **b–e**) and *Acropora hyacinthus* (**c–f**). Dashed line represents equivalence with seawater pH. Data are means ± standard deviation. For pH_ECM_ (**a–c**), three way ANOVA and simple effects analysis identified conditions where pH_ECM_ is elevated in the light with respect to dark (indicated by asterisks). For calicoblastic pH_I_ (**d**–**f**), three way ANOVA identified a significant positive effect of light on pH_I_ across the species. See Table 2 and Supplementary 2 for statistical analysis and Table 1 for carbonate chemistry corresponding to each seawater pH.

Corals were exposed to the pH treatments for 1 week, after which coral colonies of each coral species were analysed by confocal microscopy to determine pH_ECM_ in light and dark conditions (Fig. 1). pH_ECM_ values are also expressed as [H^+^] in Supplementary Fig. 3. There were significant effects of light/darkness, pH treatment and species on pH_ECM_ (Table 2 and Supplementary 2). Overall, we observed a general trend of declining pH_ECM_ with seawater acidification in all species in light and darkness. However, significant interactions were identified between species, seawater pH and light/darkness, indicating that there are differences in the way pH_ECM_ responded among the species in different conditions. In *S. pistillata*, pH_ECM_ varied from pH 8.28 ± 0.04 in light and 8.19 ± 0.05 in darkness at seawater pH 8.1, and falling to pH 7.82 ± 0.10 and 7.81 ± 0.08 at seawater pH 7.2. In this species pH_ECM_ values were not significantly different between light and darkness in any of the seawater pH treatments (Table 2 and S1). Under the irradiance used here, *S. pistillata* displayed higher values of pH_ECM_ than *P. damicornis* at seawater pH 8 and 7.2, but not *A. hyacinthus* (Supplementary 2). In darkness pH_ECM_ was higher in *S. pistillata* than the other two corals species in the seawater pH 7.4 and 7.2 treatments.

In *P. damicornis*, light pH_ECM_ fell from pH 8.15 ± 0.18 to a low pH_ECM_ of 7.65 ± 0.07. Dark values were lower, falling from pH_ECM_ 8.12 ± 0.13 to 7.51 ± 0.14. In this species pH_ECM_ was significantly elevated in the light relative to dark in the seawater pH 7.8, 7.4 and 7.2 treatments.

In *A. hyacinthus*, light pH_ECM_ fell from pH 8.18 ± 0.11 at seawater pH 8.1 to pH 7.79 ± 0.14 in seawater pH 7.2. Declines in darkness were notably much greater, dropping from pH 8.09 ± 0.14 to the lowest pH_ECM_ values measured in the investigation (pH_ECM_ 7.41 ± 0.03). Overall *A. hyacinthus* displayed the most pronounced difference in the response of pH_ECM_ to acidification between light and darkness, and pH_ECM_ was significantly different between light and dark conditions at seawater pH 7.8, 7.4 and 7.2.

### pH_I_ in the calicoblastic epithelium

All species exhibited a general trend of decreasing pH_I_ with decreasing seawater pH in both light and darkness (Fig. 1). Three-way ANOVA identified no three-way interaction between seawater pH, species and light/darkness. However, a significant two-way interaction was found to occur between seawater pH and species, indicating that calicoblastic epithelium pH responded differently to seawater pH in the three species (Supplementary 2). Light and darkness was found to have a significant effect on pH_I_, with light values higher than dark values. At the irradiance used here, the highest pH_I_ values in light and darkness were found in *S. pistillata* ranging from pH 7.47 ± 0.04 to 7.30 ± 0.02 in the light, and 7.45 ± 0.03 to 7.25 ± 0.07 in the dark. Lower values were found in *P. damicornis*, ranging from pH 7.40 ± 0.07 to 7.08 ± 0.05 in the light, and 7.36 ± 0.06 to 7.03 ± 0.07 in the dark. Even lower values were recorded in *A. hyacinthus*, ranging from pH 7.20 ± 0.06 in light to 7.04 ± 0.05, and 7.17 ± 0.01 to pH 6.93 ± 0.01 in darkness. *P. damicornis* appeared to undergo the greatest change in pHi across seawater pH treatments, however when the difference in pH_I_ between seawater pH 8.1 and 7.2 is expressed in terms of proton concentration, similar changes in [H^+^] were also observed in *A. hyacinthus* in darkness (Supplementary 3). Conversion to [H^+^] also highlights the fact that *A. hyacinthus* displayed the greatest disparity in pH_I_ of the three species between light and dark conditions in conditions of seawater acidification.

### Calcification, photosynthesis and respiration

**Figure 2:**
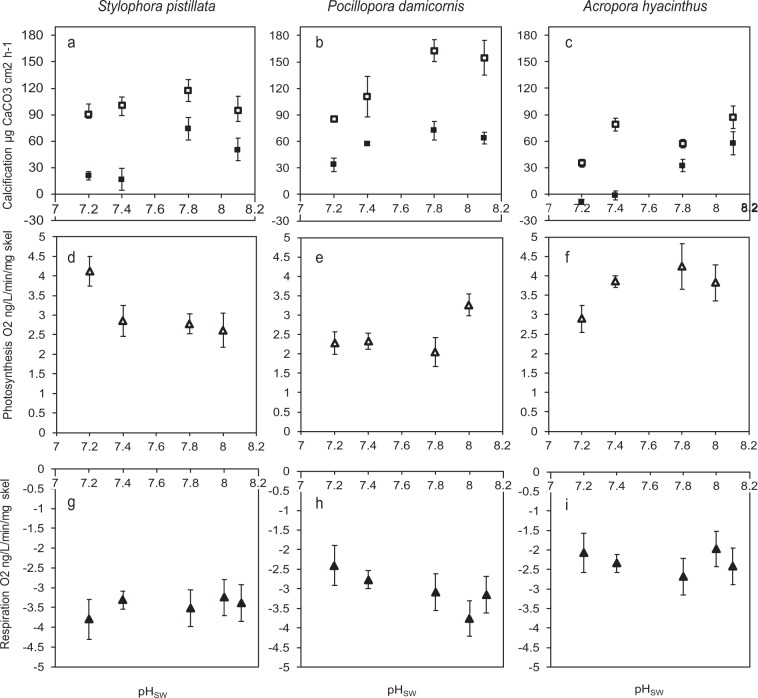
Effects of seawater acidification in light (open symbols) and darkness (closed symbols) on calcification (**a**–**c**) net photosynthetic rate (**d**–**f**) and respiration rate (**g**–**i)** in *Stylophora pistillata* (**a**–**g**), *Pocillopora damicornis* (**b**–**h**) and *Acropora hyacinthus* (**c**–**i**). Data are means ± standard deviation. See Table 2 and Supplementary 2 for statistical analysis and Table 1 for carbonate chemistry corresponding to each seawater pH.

Calcification as measured by total alkalinity anomaly was normalised by colony surface area (Fig. 2), protein and skeletal mass (Supplementary 4). The pattern of the response of calcification to acidification was very similar between the three methods of normalization. Indeed, there was no change in relationship between protein and skeletal mass across the pH treatments (Supplementary 5). Calcification rate normalized to surface area was significantly affected by species, pH treatment and light/dark conditions. Significant interactions were found between species and pH, and also species and light/dark conditions, indicating that three species responded differently to the pH treatment and the presence of light (Table 2 and Supplementary 2). No significant interaction was found between pH treatment and light/dark conditions.

In the light, calcification rates remained unchanged across the pH treatments in *S. pistillata* ranging from 95.23 ± 15.59 µg cm^−2^ h^−1^ at seawater pH 8.1 to 90.1 ± 10.94 µg cm^−2^ h^−1^ at seawater pH 7.2. However, calcification rate declined in *P. damicornis* and *A. hyacinthus*, ranging from 154.70 ± 19.55 to 85.16 ± 2.12 µg cm^−2^ h^−1^ in *P. damicornis*, and 87.30 ± 12.8 to 35.19 ± 4.38 µg cm^−2^ h^−1^ in *A. hyacinthus*. Relative to calcification rate at seawater pH 8.1, this represented a 55% and 40% decline in calcification in *P. damicornis* and *A. hyacinthus* at seawater pH 7.2 respectively.

Dark calcification rates were significantly lower than in light conditions in all species (Fig. 2; Table 2). Dark calcification rates declined in all three species with decreasing seawater pH. In *S. pistillata* and *P. damicornis*, these declines corresponded to a respective 41% and 53% decrease in calcification rate at pH 7.2 relative to pH 8.1. In *A. hyacinthus*, calcification ceased entirely at and below seawater pH 7.4 in darkness. In *A. hyacinthus* at seawater pH 7.2, increases in total alkalinity in the incubation chamber during calcification measurements indicated that dissolution occurred in this treatment in darkness.

Photosynthetic rates for the three coral species across the four seawater pH treatments are given in Fig. 2. Analysis by two-way ANOVA indicated that photosynthetic rates were significantly higher in *A. hyacinthus* than the others species (Table 2). Simple effects analysis (with Bonferroni correction) indicated that overall there was little effect of seawater pH on photosynthetic rate in the three species at the irradiance used here (Table 2), although photosynthetic rates were significantly elevated in *S. pistillata* at pH 7.2 relative to pH 8.1 (but not the other seawater pH levels).

Respiration rates also did not vary between pH treatments. However, respiration rates varied significantly between species (Table 2). Respiration rates were significantly higher in *S. pistillata* than the other two species.

## Discussion

In the current study *S. pistillata* displayed the greatest capacity to regulate pH_ECM_ against decreases in seawater pH, maintaining the highest offsets between pH_ECM_ and seawater pH in light and darkness. These *S. pistillata* pH_ECM_ measurements are consistent with previous confocal measurements of pH_ECM_ in this species at seawater pH 8 and 7.2^2^. By contrast to *S. pistillata*, both *A. hyacinthus* and *P. damicornis* displayed different responses of pH_ECM_ to acidification in light and darkness. While *A. hyacinthus* regulated pH_ECM_ to similar levels observed in *S. pistillata* in the light, it experienced pronounced decreases in pH_ECM_ in darkness. *P. damicornis* also underwent greater declines in pH_ECM_ in darkness than in the light in the acidification treatments, although the difference between light and dark treatments was less pronounced than in *A. hyacinthus*. There are two main interpretations of these findings. Firstly, that exposure to light can have a role in mitigating the impact of acidification on pH_ECM._ - this is evident in *P. damicornis* and *A. hyacinthus* in which light keeps pH_ECM_ more elevated under seawater acidification than in darkness. Secondly, as the effect of seawater acidification on pH_ECM_ varies between species, this suggests that the species investigated here vary in their capacity to regulate pH_ECM_ against seawater acidification, particularly in darkness where *S. pistillata* maintained higher pH_ECM_ than the other species. These findings are consistent with the widely held view that *S. pistillata* is a relatively resistant species to ocean acidification among coral species^26^ and with reports that Acroporid corals are physiologically less tolerant of ocean acidification^28,29^. However, comparisons of the capacity of pH_ECM_ regulation in the current study are limited by lack of knowledge of the relationship between pH_ECM_, light exposure and photosynthetic activity in the three species. Currently, the literature reports that light elevates pH_ECM_ relative to dark levels (e.g. increases in pH_ECM_ in the light have been observed previously both in *S. pistillata* and *Galaxea fasicularis* at seawater pH 8.1^4,7^), but it is not known what level of photosynthetic activity is required to reach maximum levels of pH in ECM. Furthermore, photosynthesis-irradiance curves were not conducted in the current study and thus it is not known whether coral photosynthesis was operating at maximum or lower rates in the three species under the irradiance used here. As such we cannot rule out the possibility that the relative performance of the coral species may differ under a different irradiance regime. This clearly constitutes an important area for further research.

Measurements of calicoblastic cell pH_I_ in the acidification treatments revealed a similar pattern of response to pH_ECM_ among the species, suggesting that acid-base regulation of the ECM and calicoblastic cells is closely linked. *S. pistillata* maintained highest pH_I_ in light and darkness, and greater declines in pH_I_ were observed in *P. damicornis* and *A. hyacinthus*. The largest differences between light and dark pH_I_ were observed at low pH in *A. hyacinthus*, consistent with what was observed for pH_ECM_. Interestingly, analysis of pHi data by three-way ANOVA also revealed a small but significant, positive effect of light on calicoblastic pH_I_ (in the range of 0.02-0.1 pH units). This was most apparent in *A. hyacinthus* in the lower seawater pH treatments (Fig. 1). This is surprising because previous research on isolated cells and coral microcolonies suggests that light increases pH_I_ only in coral endoderm cells harbouring photosynthetic symbionts^23–25^. Indeed, previous work with *P. damicornis* has shown that light-driven increases in pHi of endoderm cells may help protect against acidosis at low seawater pH^25^. Here in the current study our findings suggest that light can also drive pHi increases in cells of the calicoblastic cell layer which doesn’t contain symbionts, but is adjacent to the endoderm layer. The light-driven shifts in calicoblastic cell pH_I_ are much smaller in comparison to those observed in endoderm cells (which occur in the range of 0.3–0.4 pH units^24^). Light effects on calicoblastic pH_I_ may not have been observed previously because light/dark comparisons were carried out at pH 8.1^4^, and the increases in pH_I_ observed here are most apparent at lower seawater pH. Also, these previous measurements of calicoblastic cells were carried out with a different experimental design and may not have had the statistical power to distinguish the small light and dark differences. In any case, in accordance with further research proposed above for the relationship of pH_ECM_ and photosynthetic activity, additional work is required to better characterize the effect of light on pHi regulation in intact corals in both the calicoblastic cells and other tissue layers.

Understanding of the mechanistic basis underlying potential differences in coral species to regulate pH_ECM_ and pHi in light and darkness is limited. Previous work with cnidarian endoderm cells invokes the role of Na^+^/H^+^ exchangers^40^. In calicoblastic cells, a Ca^2+^ATPase and a HCO_3_^−^/Cl^−^ exchanger have been localized in *S. pistillata*, and have thus been proposed to have roles in acid-base regulation linked to calcification^41,42^. However, mechanisms of pH regulation are likely to vary between species. A previous study that identified differences between *P. damicornis* and *Montipora capitata* in the capacity to regulate pHi at elevated temperature suggests there are links between mechanisms of thermotolerance and pHi regulation^43^. Other research indicates a differential localization of transporters with possible roles in calcification in *Acropora yongeii* compared to *S. pistillata*^44^. For example, this latter study indicates an abundance of Na^+^/K^+^-ATPase in the apical membrane of the oral epithelium in *A. yongei* but not *S. pistillata*, while Ca^2+^ATPase was abundant in the endoderm of *S. pistillata* but not *A. yongei*. Additionally several proposed mechanisms explaining the role of light in elevating pH_ECM_ have been published previously^4,7,45^. Among these it has been suggested that pH increases in the coelenteron lumen due to symbiont photosynthesis may provide a favourable gradient for the removal of protons from the ECM^46,47^. In the case of the calicoblastic cells, it could be hypothesized that increases in coelenteron pH and the adjacent endoderm cell layer may also promote the movement of protons from the calicoblastic cells resulting in slight increases in calicoblastic pH_I_ observed in the current study. As such, light-driven increases in pHi that may help endoderm cells buffer against seawater acidification may also mitigate decreases in pHi in calicoblastic cells. However, as it stands, little functional data are available for proton transport in corals or the role of ion transporters localized to the calicoblastic cells, and this is an essential avenue for future research to gain a better mechanistic understanding of calcification.

We conducted parallel experiments to gain insight into how acidification in light and darkness affects coral photosynthesis, respiration and calcification, as these metabolism parameters could potentially influence pH regulation. Generally, no significant effect of seawater acidification was observed on photosynthetic rates in the three species at the irradiance level used here, although photosynthetic rates were significantly elevated at seawater pH 7.2 relative to pH 8 in *S.pistillata*. Although elevated pH_ECM_ in light relative to dark conditions in *P. damicornis* and *A. hyacinthus* clearly indicates that photosynthesis has a positive effect on pH_ECM_ under acidification in these species, the general lack of response of photosynthetic rates in the three species observed here makes it difficult to attribute a role for this parameter in determining the relative sensitivity of pH_ECM_ and pH_I_ to acidification. However, as we state above, it is necessary to fully characterize the photosynthesis-irradiance (PI) response of each species in each treatment before firm conclusions about the response of photosynthesis and its role in pH regulation can be made. In the meantime, we note that the insensitivity of photosynthesis observed here in the three species is in agreement with previous work on *S. pistillat*a at the same irradiance used here^2^, and also meta-analysis carried out by Kroeker *et al*.^48^ conducted on eleven investigations of seawater acidification on a range of coral species which indicated that seawater pCO_2_ had no overall significant effect on photosynthesis. However the influence of seawater acidification on coral photosynthesis can be equivocal, with both negative^49,50^ and positive effects^51^ also having been reported.

Respiration rates could also be relevant to pH regulation, because higher rates of CO_2_ production could potentially present a greater challenge to acid-base regulation of both calicoblastic cells and the calcifying medium. In this respect, if seawater acidification caused higher respiration rates in the corals, then negative effects on pH regulation might be expected. Again this was not the case here, as respiration rates did not significantly change between the pH treatments in the three species. Similar to photosynthesis, previously reported responses of coral respiration to seawater acidification can also be rather equivocal. Decreases in respiration rate under elevated pCO_2_ have been reported for *Acropora millepora* and massive *Porites* sp. corals, but a recent study by^52^ observed no effect of pCO_2_ on respiration in six (including *P. damicornis*) out of 8 species tested, in agreement with what was observed here in the current study.

In the current study, effects of seawater acidification were much more apparent on calcification rates than the other metabolism parameters. Generally, increasing seawater acidification had a negative effect on calcification, but the effects were species-specific and influenced by light and darkness. All three species exhibited light-enhanced calcification (LEC), a phenomenon that has been observed in numerous studies on corals^20^. In *S.pistillata* light calcification rates were unaffected by seawater acidification, however in darkness, calcification rates were significantly lower at seawater pH 7.4 and pH 7.2 relative to pH 8. In *P. damicornis*, light calcification rates also decreased under acidification, while dark calcification rates did not, and in *A. hyacinthus* both light and dark calcification decreased in acidification treatments. As calcification- irradiance response curves were not carried out during this investigation, it is not known whether these observed responses occurred at optimum light levels for calcification. However, the current data do indicate that calcification is more sensitive to acidification in darkness in *S. pistillata* and *A. hyacinthus* which is in line with previous studies in the literature^21,53^. Interestingly in *A. hyacinthus*, calcification ceased entirely in the dark at pH 7.4 and dissolution of the skeleton occurred at pH 7.2 in darkness. This is consistent with reports of skeletal dissolution in the coral *Acropora millepora*^53^ in darkness under elevated pCO_2_ conditions (1073uatm).

pH_ECM_ and calcification rates are anticipated to be linked, because increases in pH_ECM_ would be expected to increase the relative proportion of [CO_3_^2−^] in the dissolved inorganic carbon pool and therefore Ω of the ECM thereby favouring calcification. Broadly, calcification rates in the three species followed a similar trend to pH_ECM_, as calcification rates declined as pH_ECM_ decreased with increasing seawater acidification. The most dramatic declines in calcification rate were observed in darkness in *A. hyacinthus* which also exhibited that largest declines in pH_ECM_. In the case of *P. damicornis* and *A. hyacinthus*, both pH_ECM_ and calcification rates were lower in darkness than in the light. These data fit the view that pH_ECM_ influences rates of skeleton formation^4,15,54^. There are however a number of inconsistencies between pH_ECM_ and calcification rate, suggesting the relationship between pH_ECM_ and calcification is more complex than previously thought. In *P. damicornis*, pH_ECM_ declined significantly between pH 8.1 and 7.8 in light and darkness, but no accompanying change was observed in calcification rates. We also made the same observation in *S. pistillata* with pH_ECM_ declining between the 8.1 and 7.8 treatments in light and dark, but with no accompanying decrease in calcification. Other interspecies comparisons have also shown how variation in coral calcification rates can bear little relation to changes pH_ECM_ in corals exposed to changes in seawater carbonate chemistry, including a recent study by Comeau *et al*. that used boron isotope systematics to determine pH_ECM_ on *P. damicornis* and another Acroporid (*A. yongei*)^54^.

The *in vivo* approach used in the current study allowed us to explore the effect of light and darkness on the relationship between pH_ECM_ and calcification rate, something that was not possible in the recent boron isotope study on *P. damicornis* and *A. yongei*^54^. We observed in *S. pistillata*, that although calcification rates were consistently higher in the light versus the dark both at pH 8.1 and in conditions of acidification down to seawater pH 7.2, light and dark pH_ECM_ were not different. By illustration, at seawater pH 7.4, light calcification was 10 times higher than dark calcification, but light and dark pH_ECM_ are identical. These data do not support the previously proposed idea that light-driven elevation of pH_ECM_ is involved in the mechanism underlying light enhanced calcification (LEC), at least in the case of *S. pistillata*^4^.

The difficulties in reconciling pH_ECM_ and calcification data in light and darkness maybe related to the fact that the pH_ECM_ measurements and calcification measurements were performed on different experimental material (i.e. microcolonies on glass coverslips and microcolonies suspended on thread). There may be differences in tissue thickness, symbiont densities and rates of exchange between ions in ECM and seawater in these two different types of microcolony preparation, which could influence their physiological response to acidification and light. Alternatively, inconsistencies between pH_ECM_ and calcification rate may arise from the fact that calcification is ultimately driven by numerous physiological factors in addition to pH_ECM_ which may also be responsive to acidification. Indeed recent research highlights three aspects of calcification physiology that could influence the calcification response to acidification: regulation of Ca^2+^ concentration in the ECM^55,56^, dissolved inorganic carbon concentration [DIC] in the ECM^57^ and the role of the organic matrix in the biomineralization process^20^. In the case of Ca^2+^ regulation, recently developed geochemical approaches indicate that maintenance of calcification rates under acidification may be related to the capacity of corals to elevate [Ca^2+^] in the ECM^55^. In this study, an acidification-tolerant *P. damicornis* was found to elevate [Ca^2+^] in the ECM with respect to seawater, whereas an acidification-sensitive *A. youngei* did not. It is therefore plausible that a differential ability to regulate ECM [Ca^2+^] in the corals of the present study may have influenced calcification rates. Turning to ECM [DIC], this parameter (together with [Ca^2+^] and pH) determines ECM Ω and could therefore influence calcification^16,55,57,58^. While recent geochemical studies indicate that resistance to acidification may only involve moderate increases in ECM [DIC]^59^, further research into the role of ECM DIC regulation in determining calcification rates is needed. Lastly, a growing body of research is elucidating the role of the organic matrix (OM) (which consists of proteins, sugars and lipids) in coral calcification. OM components have several proposed roles in the calcification mechanism including reducing the free energy required for aragonite crystal nucleation^60^ and controlling the growth of aragonite crystals^20,61–63^. As such, the production of the OM may also have a key role in determining calcification rates under acidification^62^. Indeed, previous research on *S. pistillata* indicates that this coral species increases OM production under acidification, possibly to promote calcification under less favourable conditions^2^. In addition to its role in calcification, the presence of organic matrix in the skeleton has also been reported to influence its solubility^64,65^, suggesting that interspecies differences in the composition and/or quantity of organic matrix may also affect the tendency of skeletons of certain species to undergo dissolution before others. This may explain why in the current study at seawater pH 7.2, we observed skeletal dissolution in darkness in *A. hyacinthus* and not *P. damicornis*, although both species maintained a similar pH_ECM_ (pH 7.5-7.4).

Overall, it is therefore likely that the response of coral calcification to acidification may depend on several interacting aspects of calcification physiology in addition to pH regulation^54,55,58,62,63^. The interspecies differences identified in the current work suggest that mechanisms of calcification physiology may vary among scleractinians. One question is whether the different responses observed here were influenced by scleractinian phylogeny. Robust and complex clades are thought to have evolved calcification independently^30^ and thus ion transport mechanisms linked to calcification (such as those involved in pH_ECM_ regulation) might be expected to be different between these groups^44^. *S. pistillata* and *P. damicornis* are members of the robust clade, while *A. hyacinthus* belongs to the complex clade of scleractinia. The susceptibility of both pH regulation and calcification to acidification in *A. hyacinthus* does indeed suggest that this species is the outlier of the group. Furthermore, even under ambient seawater pH 8.1 conditions, *A. hyacinthus* displayed a markedly lower intracellular pH suggesting that its system of cellular acid-base balance is distinct from the other species. On the other hand, the pattern observed here does not easily correspond to the robust and complex groups, because both robust members (*S. pistillata* and *P. damicornis*) exhibit distinct responses themselves. As such, future investigation of more members of each clade is required before clear conclusions about clade-specific physiological traits can be made. Similarly, future work could investigate the potential influence of different symbiont types (i.e. different members of the family *Symbiodiniaceae*^18^) associated with the three coral species on their pH regulation. If the coral species investigated here associate with symbiont types that vary in their response to light and seawater acidification, then differences in symbiont communities in each coral may also influence pH regulation and the physiology of each coral species under acidification. Additionally, deciphering mechanistic differences in pH regulation and calcification between the species may be achieved using manipulations of seawater carbonate chemistry beyond what was performed in the current investigation. Here, we chose to investigate the impact of seawater acidification by CO_2_ enrichment due to its environmental relevance to climate change, but future work could potentially reveal interesting mechanistic differences by manipulating seawater bicarbonate and carbonate concentrations in a manner similar to some previous investigations^17,45^.

In summary, the current study reveals differences in the extent to which pH_ECM_, pH_I_ and coral calcification are impacted by acidification in light and darkness. An important caveat of our study was that it was conducted under controlled, laboratory conditions (which has advantages for mechanistic research), at a single light level and as such, the current data cannot easily be extrapolated to the performance of these corals in the field where numerous environmental parameters vary in concert. An important future challenge will be to use what we learn in the laboratory to orientate field-based investigations into coral calcification physiology in the natural environment. This will be an essential step in understanding the adaptive capacity of corals to a changing ocean, both in terms of their geological past and their future in coming decades.

## Supplementary information

SUPPLEMENTARY INFORMATION
