# Supplementary material for: Effects of light and darkness on pH regulation in three coral species exposed to seawater acidification
Source: Sci Rep. 2019 Feb 18;9:2201. doi: 10.1038/s41598-018-38168-0 (PMC6379376; doi:10.1038/s41598-018-38168-0)

SUPPLEMENTARY INFORMATION

Effects of light and darkness on pH regulation in three coral species exposed to seawater acidification

Venn AA[1,2*], Tambutté E[1,2], Caminiti-Segonds N [1,2], Techer N [1,2], Allemand D [1,2], Tambutté S [1,2].

1. Marine Biology Department, Centre Scientifique de Monaco, 8 Quai Antoine 1er, Monaco 98000

2. Laboratoire Européen Associe 647 « BIOSENSIB », Centre Scientifique de Monaco-Centre National de la Recherche Scientifique, 8 Quai Antoine 1er, Monaco 98000

*****Corresponding authors email + 377 97 77 44 72 avenn@centrescientifique.mc

Supplementary 1. pH (mean ± SD) in the seawater surrounding the corals in the perfusion chamber during confocal analysis.

pH Treatment		*Stylophora pistillata*		*Pocillopora damicornis*		*Acropora hyacinthus*	
		Mean	SD	Mean	SD	Mean	SD
8.1	Light	8.07	0.06	8.08	0.09	8.07	0.06
	Dark	8.08	0.07	8.11	0.07	8.10	0.02
7.8	Light	7.81	0.05	7.80	0.06	7.88	0.08
	Dark	7.85	0.07	7.80	0.07	7.85	0.01
7.4	Light	7.43	0.02	7.41	0.02	7.44	0.09
	Dark	7.44	0.02	7.40	0.04	7.38	0.05
7.2	Light	7.23	0.15	7.23	0.02	7.21	0.05
	Dark	7.24	0.06	7.22	0.02	7.26	0.06

Supplementary 2. Results of simple effects analysis on significant interactions identified by three way ANOVA in Table 2 in the main manuscript. SP = *Stylophora pistillata*; PD = *Pocillopora damicornis*; AH = *Acropora hyacinthus*.

Simple effects for pH_{ECM}

pH_{ECM}	L/D	pH_{SW}	Species
	Light	8.1	SP=AH; SP>PD
		7.8	ND
		7.4	ND
		7.2	SP>PD; AH>PD; SP=AH
	Dark	8.1	ND
		7.8	ND
		7.4	SP>PD; SP>AH; PD=AH
		7.2	SP>PD; SP>AH; PD=AH

pH_{ECM}	pH_{SW}	Species	L/D
	8.1	ST	ND
		PD	ND
		AH	ND
	7.8	ST	ND
		PD	Light > Dark
		AH	Light > Dark
	7.4	ST	ND
		PD	Light > Dark
		AH	Light > Dark
	7.2	ST	ND
		PD	Light > Dark
		AH	Light > Dark

pH_{ECM}	L/D	Species	pH_{SW}
	Light	ST	8.1> 7.8; 8.1>7.4; 8.1>7.2; 7.8>7.4; 7.4=7.2
		PD	8.1> 7.8; 8.1> 7.4; 8.1> 7.2; 7.8> 7.4; 7.8> 7.2; 7.4=7.2
		AH	8.1> 7.8; 8.1> 7.4; 8.1> 7.2; 7.8> 7.4; 7.8> 7.2; 7.4=7.2
	Dark	ST	8.1> 7.8; 8.1>7.4; 8.1>7.2; 7.8=7.4; 7.8=7.4; 7.4=7.2
		PD	8.1> 7.8; 8.1> 7.4; 8.1> 7.2; 7.8> 7.4; 7.8> 7.2; 7.4=7.2
		AH	8.1> 7.8; 8.1> 7.4; 8.1> 7.2; 7.8> 7.4; 7.8> 7.2; 7.4=7.2

Simple effects for pH_i

pHi	Species	pH_{SW}
	ST	8.1>7.4,7.2; 7.8>7.4,7.2; 7.4>7.2; 8.1=7.8
	PD	8.1>7.4,7.2; 7.8>7.4,7.2; 7.4>7.2; 8.1=7.9
	AH	8.1>7.8,7.4,7.2; 7.8>7.4,7.2; 7.4=7.2
**pHi**	**pH_{SW}**	**Species**
	8.1	ST> PD; ST> AH; PD> AH
	7.8	ST> PD; ST> AH; PD> AH
	7.4	ST> PD; ST> AH; PD> AH
	7.2	ST> PD; ST> AH; PD> AH

Simple effects for Calcification Rate

Calcification rate	L/D	pH_{SW}	Species
	Light	8.1	PD> SP; PD> AH; SP =AH
		7.8	PD>SP; PD>AH; SP>AH
		7.4	SP,PD>AH; SP=PD
		7.2	SP> AH, PD> AH; SP=PD
	Dark		
		8.1	ND
		7.8	SP, PD >AH; SP=PD
		7.4	PD> SP; PD> AH; SP =AH
		7.2	SP, PD > AH; SP=PD
**Calcification rate**	**pH_{SW}**	**Species**	**L/D**
	8.1	ST	Light > Dark
		PD	Light > Dark
		AH	Light > Dark
	7.8	ST	Light > Dark
		PD	Light > Dark
		AH	Light > Dark
	7.4	ST	Light > Dark
		PD	Light > Dark
		AH	Light > Dark
	7.2	ST	Light > Dark
		PD	Light > Dark
		AH	Light > Dark
**Calcification rate**	**L/D**	**Species**	**pH_{SW}**
	Light	ST	ND
		PD	8.1 > 7.4, 7.2; 7.8 > 7.4,7.2; 8.1= 7.8; 7.4 =7.2
		AH	8.1, 7.8, 7.4 > 7.2; 8.1=7.8;8.1=7.4;7.8=7.4
	Dark	ST	8.1>7.4; 7.8> 7.4,7.2; 7.4= 7.2
		PD	7.8> 7.2; 8.1= 7.4, 7.8, 7.2; 7.4=7.2
		AH	8.1>7.4, 7.2; 7.8>7.4,7.2; 8.1 =7.8; 7.4=7.2;

Supplementary 3. Effects of seawater acidification in light (open symbols) and darkness (closed symbols) on proton concentration [H^+] in the extracellular calcifying medium (a-c) and calcifying cells (d-f) in *Stylophora pistillata* (column a-g), *Pocillopora damicornis* (column b-h) and *Acropora hyacinthus* (c-i). Data are means ± standard deviation.

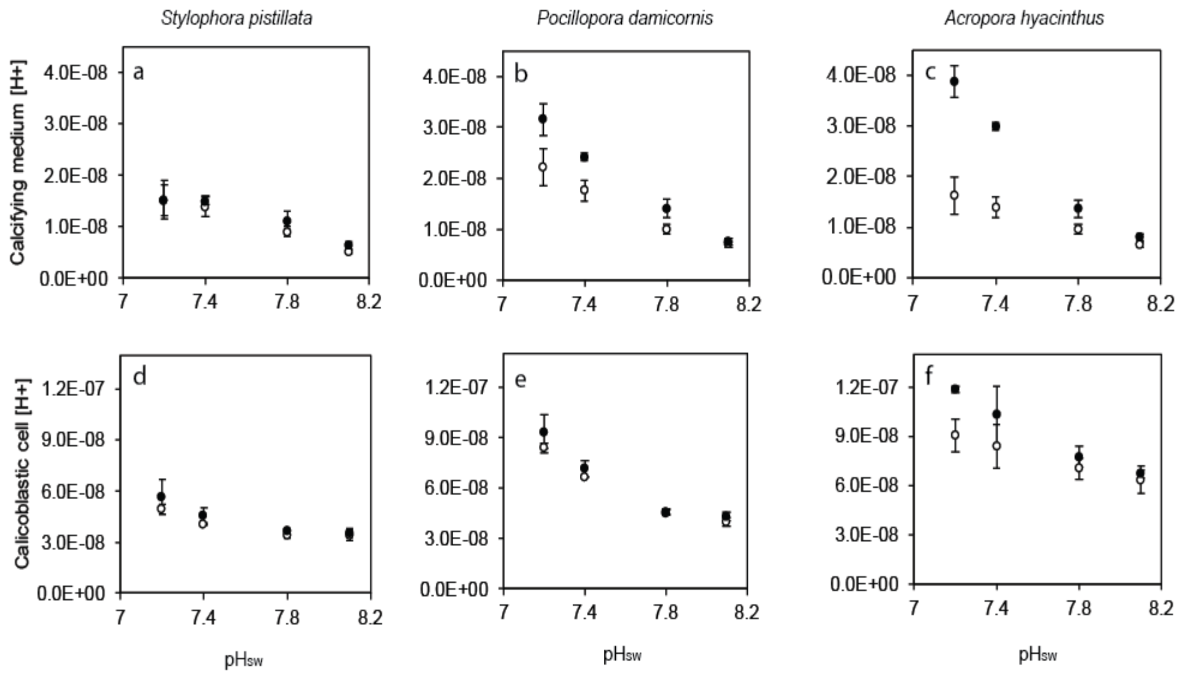

Supplementary 4. Effects of seawater acidification in light (open symbols) and darkness (closed symbols) on calcification rates normalized to protein (a-c) and skeletal mass (d-f). *Stylophora pistillata* (column a-d), *Pocillopora damicornis* (column b-e) and *Acropora hyacinthus* (c-f). Data are means ± standard deviation.

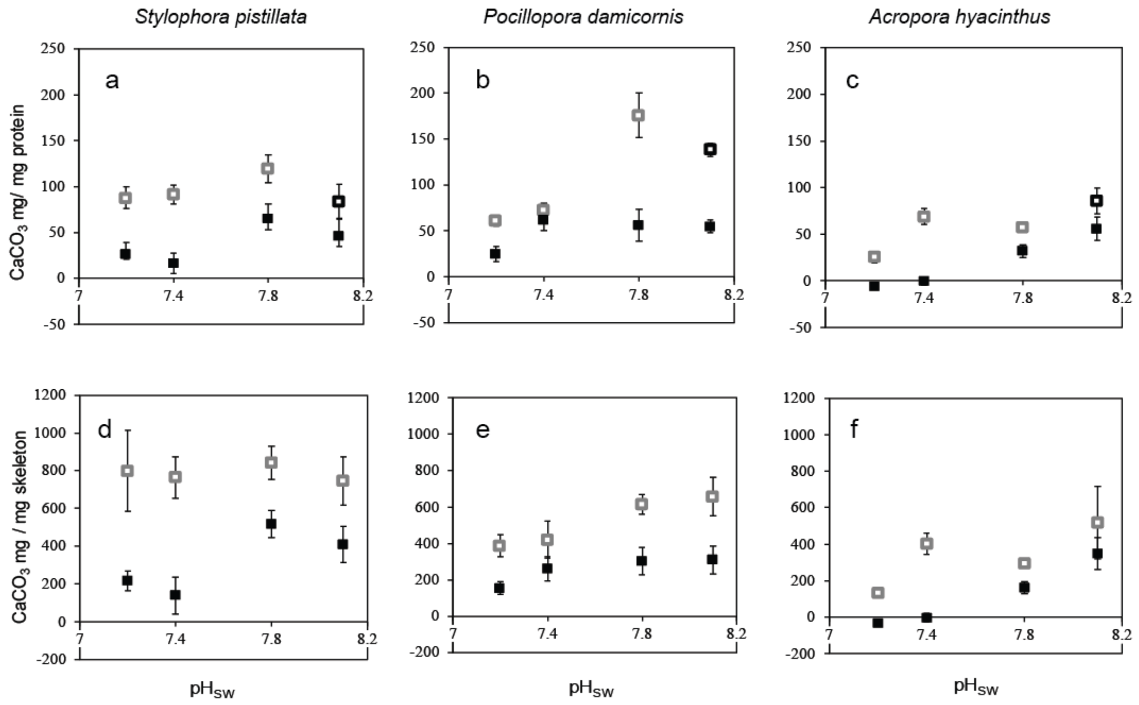

Supplementary 5. The ratio between protein and skeletal mass in the four pH treatments. Data are means ± standard deviation.

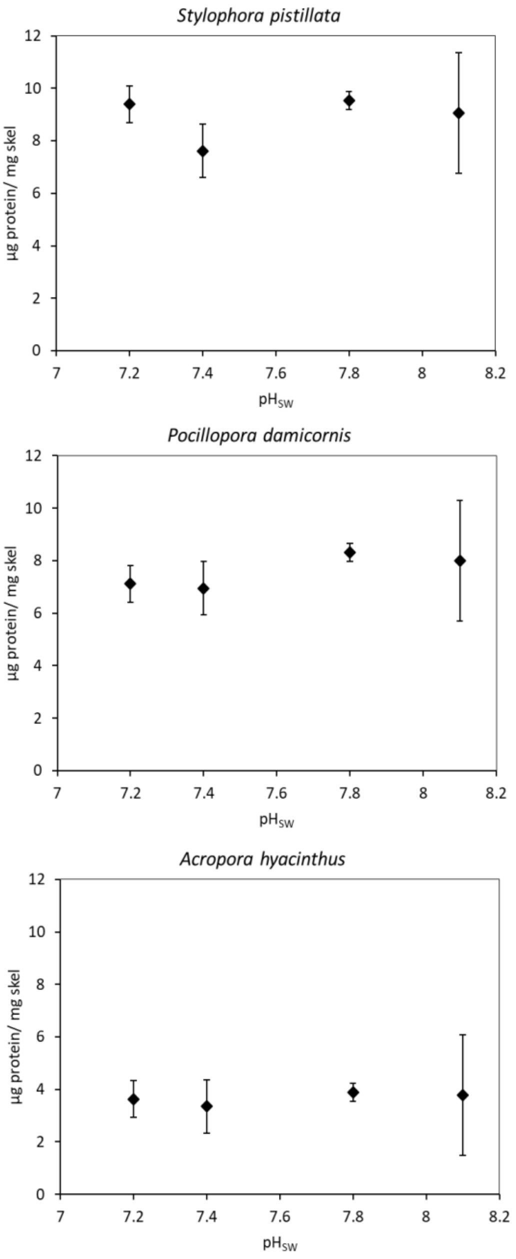

Supplement: Supplementary file 1 — SUPPLEMENTARY INFORMATION [file 41598_2018_38168_MOESM1_ESM.pdf]
